# Caudal Duplication Syndrome in an Asymptomatic Primi Postnatal Patient

**DOI:** 10.7759/cureus.32773

**Published:** 2022-12-21

**Authors:** Ayesha Afridi, Fauzia Afridi, Zainab Afridi, Ashfaq Afridi

**Affiliations:** 1 Radiology, Federal Government Polyclinic Hospital, Islamabad, PAK; 2 Obstetrics and Gynecology, Khyber Teaching Hospital/Khyber Medical University, Peshawar, PAK; 3 Radiology, Government Maternity Hospital Hashanagar, Peshawar, PAK; 4 Anesthesia and Intensive Care, Cork University Hospital, Cork, IRL

**Keywords:** neural system, spinal, genitourinary tract, gastrointestinal tract, malformations, genetic disorders, anomaly scan, conjoined twinning, postnatal, caudal duplication syndrome

## Abstract

Caudal duplication syndrome (CDS) is a rare congenital anomaly in which a wide spectrum of malformations ranging from partial or isolated to complete duplication of caudal organs in the gastrointestinal tract (GIT), genitourinary tract (GUT), and spinal and neural systems occur. Its exact cause is unknown, however various factors such as genetic disorders and conjoined twinning are mentioned in the etiology of CDS. Second-trimester anomaly scan can diagnose this anomaly prenatally.

This case report describes a primi postnatal patient with CDS without any neurological symptoms. She gave birth to a healthy baby girl by cesarean section with breech presentation as an indication.

## Introduction

Duplication of the genitourinary system, colon, distal spine, spinal cord, and lower limb is a complex and very rare congenital condition known as “caudal duplication syndrome” (CDS). This syndrome was first described by Dominguez et al. [1]. It is sometimes referred to as a type of incomplete separation of mono-ovular twins or conjoined twinning [2]. Certain hypotheses are proposed to describe the etiology of CDS, comprising incorrect expression of homeobox (HOX) genes, an initial trauma to the urorectal septum and further atypical degeneration or duplicating processes that disturb the embryological process [3].

Varying degrees of duplication of the external genitalia, cervix and uterus, bladder, colon, anus, distal spine, and limbs [4] are shown in case reports. Apart from duplication abnormalities, meningomyelocele, vertebral segmentation anomalies, omphalocele, umbilical hernia, and imperforate anus have been discussed by some authors [5]. The estimated prevalence of this syndrome is lesser than 1 per 100,000 births [6] and as compared to males, this syndrome seems to be more common in females 2:1 without any familial or racial preferences [7]. Female patients are usually infertile or have a history of repeated miscarriages [8]. Only one case was reported by Ragab et al. who attained full-term pregnancy [9].

## Case presentation

Here we report a case of a 28-year-old postnatal patient from a low- to middle-class background. She came to the radiology department on the third day of her first delivery by cesarean section due to breech presentation. She was sent for an ultrasound scan to rule out post-cesarean section pelvic collection, which was actually a double urinary bladder.

An ultrasound scan revealed uterine didelphys with a left-sided postnatal bulky uterus and a right-sided non-gravid uterus. Furthermore, two separate cervices, vaginal canals, urinary bladders, and rectum were seen. She had mild left-sided hydronephrosis with proximal hydroureter. The right kidney and ovaries were unremarkable.

**Figure 1:**
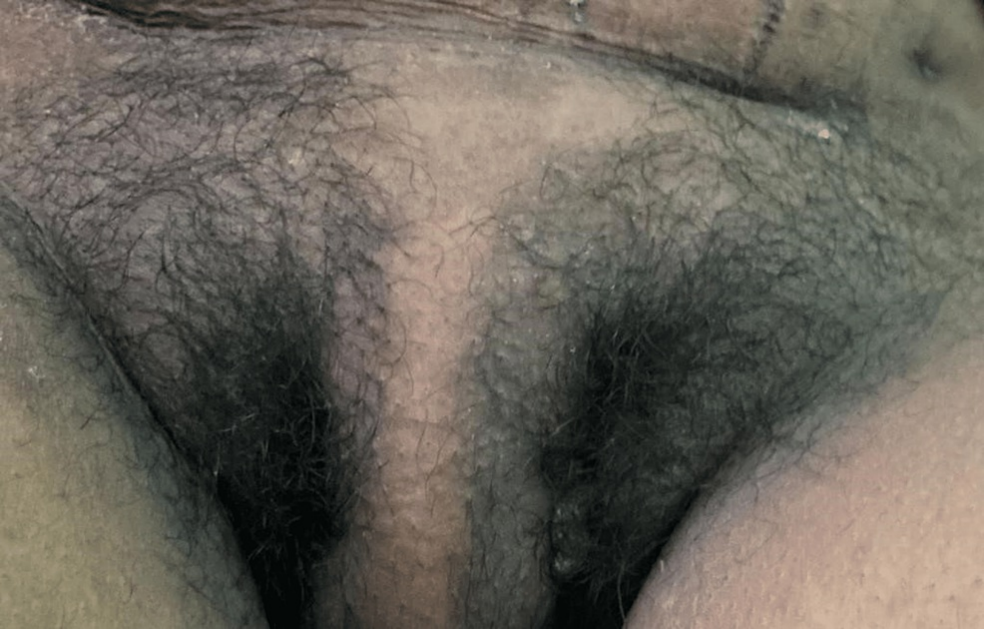
Duplication of external genitalia

**Figure 2:**
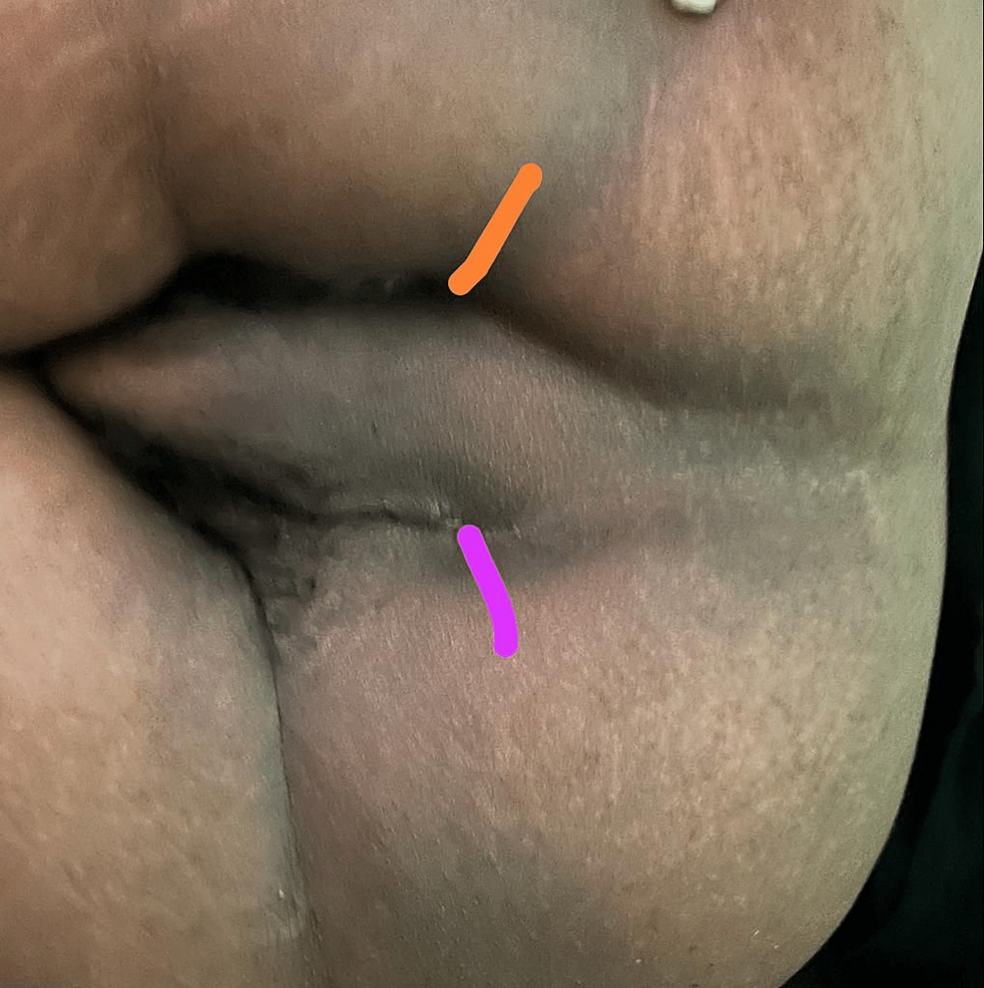
Duplication of anal orifices (marked) and buttock clefts

On clinical examination, she had duplicated external genitalia (Figure 1) and anal openings (Figure 2). There were two buttock clefts with a pad of fat in between. Each buttock cleft showed one anal opening. She had two vaginas with three labias, one labia lateral to each vagina and the third fold of labia in between the two. She confirmed both urethral openings and anal openings to be functional. She menstruated through both vaginal orifices; however, she exhibited no detrimental effects. Her lower limbs were unremarkable. Her post-operative notes showed a left-sided gravid uterus with a right-sided non-gravid uterus. Her antenatal checkups were infrequent and were mostly unremarkable till her delivery time.

**Figure 3:**
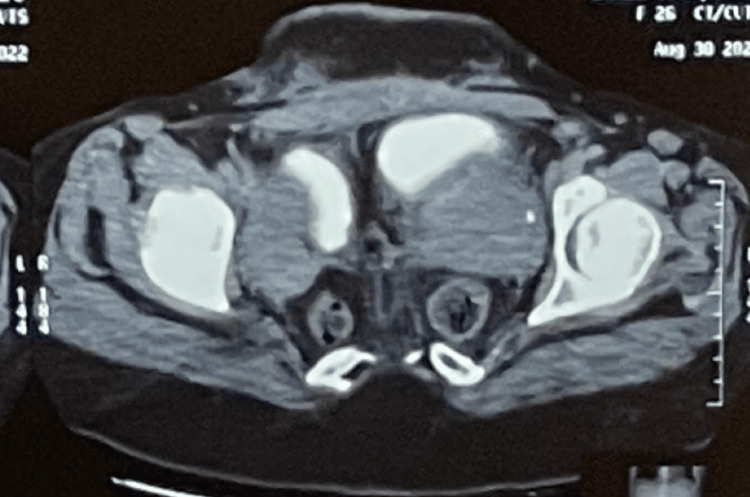
CT scan of pelvis showing duplication of pelvic viscera CT: computed tomography

**Figure 4:**
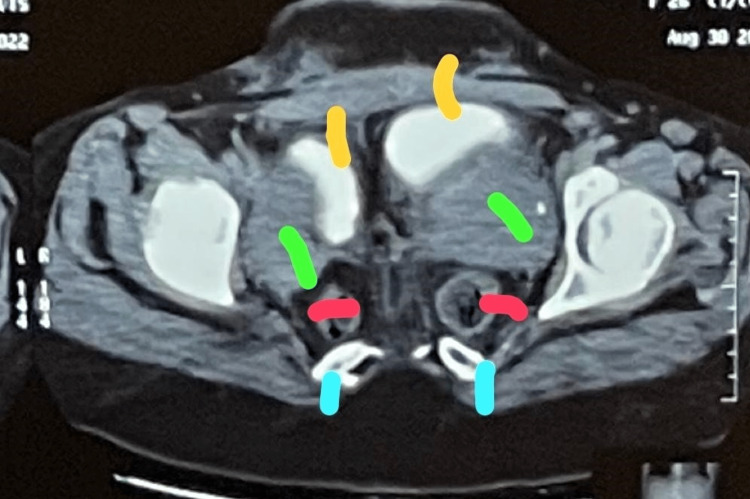
Annotated image. Yellow lines show two urinary bladders, green lines show two uteruses, red lines show two anal canals, and blue lines show two sacrums

**Figure 5:**
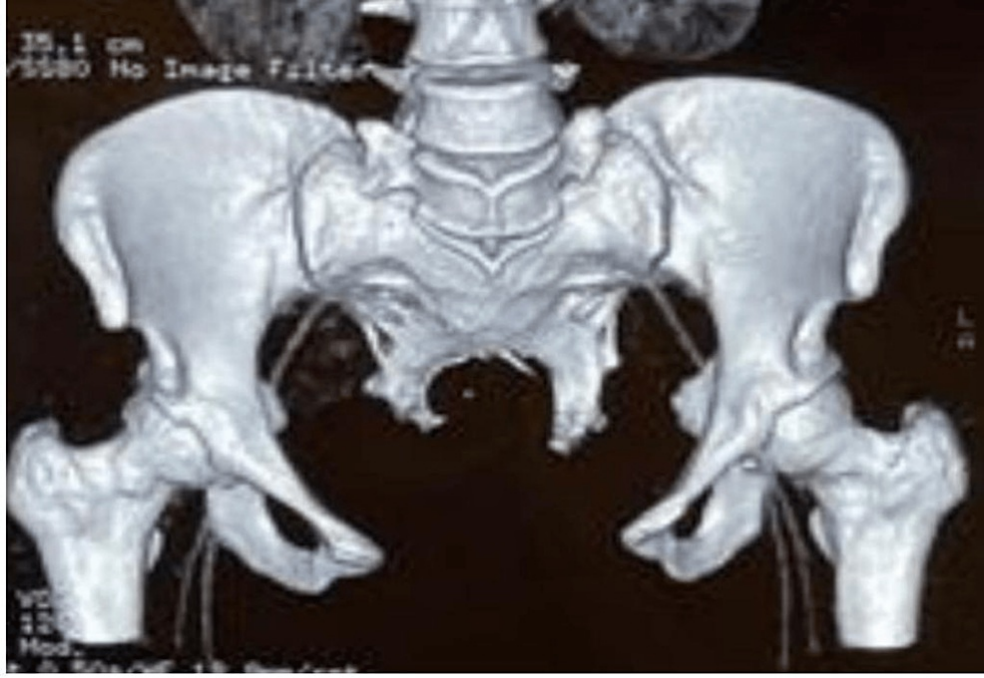
3D CT scan showing segmentation of first sacral vertebra, duplication of sacrum and pubic diastasis CT: computed tomography

Her computed tomography (CT) scan abdomen and pelvis confirmed duplicated colon, rectum, anal canals, uterus (Figure 3), cervix, vagina, urinary bladder (Figure 4), urethra, and coccyx. Also, there was segmentation of LV5 and sacrum and diastasis of the pubic bone (Figure 5). The case is unique in that the patient never underwent any reconstructive surgery nor did she have any neurological deficits or symptoms.

## Discussion

CDS is a rare congenital anomaly with only about 42 cases of CDS documented in literature till 2018 [10]. However, in Wang K et al. studies, a total of 51 patients including three from their team were selected for their research [11]. The pathogenesis of CDS is unclear. It is attributed to the incomplete separation of mono-ovular twins, which helps explain, as in this case, duplication of the spine, hindgut, and lower genitourinary tract (GUT) [12]. This female patient showed a case of duplicated organs involving the gastrointestinal tract (GIT) and GUT and anomalies of the distal vertebral column with preserved fertility. The cloaca of the embryo develops during the third to fourth week of gestation. It forms a common channel that gives rise to the GUT and GIT from the anterior and posterior portions respectively as the urorectal septum migrates caudally [13]. The common embryological origin describes the frequent association of anomalies involving these systems.

These patients are initially assessed at their birth because of obvious perineal features. Because of the low incidence of CDS and less awareness about this entity, an antenatal examination can detect a group of severe anomalies but might not recognize this as CDS [1]. Most cases of CDS are brought for treatment/surgical management in infancy or early childhood. The quality of life in CDS patients may be affected in many aspects due to the complex malformations of the caudal organs. This case is special in that it offers a unique perspective of CDS, the patient who did not undergo reconstructive surgery. She had a normal full-term pregnancy, a normal cesarean section, and a healthy baby.

## Conclusions

In a nutshell, very little is written in literature about this rare syndrome. Extensive diagnostic workup may be required in some cases to analyze the anatomy of CDS patients which will help in the proper management of the patient. Investigations like ultrasound, x-ray radiography, barium enema, micturating cystourethrogram, CT scan, and MRI scan should be carried out. CDS is a rare entity and a lot of research work is needed to be done for a proper understanding of its etiology, diagnosis, management, and awareness.
